# Assessment of interventions to attract and retain health workers in rural Zambia: a discrete choice experiment

**DOI:** 10.1186/s12960-019-0359-3

**Published:** 2019-04-03

**Authors:** Margaret L. Prust, Aniset Kamanga, Lupenshyo Ngosa, Courtney McKay, Chilweza Musonda Muzongwe, Mazuba Tamara Mukubani, Roy Chihinga, Ronald Misapa, Jan Willem van den Broek, Nikhil Wilmink

**Affiliations:** 1 Applied Analytics Team, Clinton Health Access Initiative, Inc., 383 Dorchester Ave., Suite 400, Boston, MA 02127 United States of America; 2 Human Resources for Health Team, Clinton Health Access Initiative, Inc., Lusaka, Zambia; 3 Planner-Development Cooperation, Ministry of Health, Lusaka, Zambia; 4 Human Resource and Administration, Ministry of Health, Lusaka, Zambia; 5 Human Resource and Administration, Zambia Public Sector Management Division, Lusaka, Zambia; 6 Clinton Health Access Initiative, Inc., Lusaka, Zambia

**Keywords:** Human resources for health, Zambia, Rural retention, Discrete choice experiment

## Abstract

**Background:**

Workforce shortages, particularly in rural areas, limit the delivery of health services in Zambia. Policymakers and researchers co-created this study to identify potential non-monetary employment incentives and assess their cost-effectiveness to attract and retain public sector health workers to the rural areas of Zambia.

**Methods:**

The study consisted of two key phases: a discrete choice experiment (DCE), preceded by a qualitative component to inform DCE questionnaire development. Firstly, in qualitative interviews with 25 health workers and focus group discussions (FGDs) with 253 health students, participants were asked to discuss job attributes and potential incentives that would influence their job choices. Based on this exercise and in consultation with policymakers, job attributes were selected for inclusion in a discrete choice experiment (DCE) questionnaire. Secondly, this questionnaire, consisting of hypothetical job “choice sets,” was presented to 474 practicing health workers and students. A conditional logit regression model was applied to the data from this DCE questionnaire to estimate preferences for various job attributes. Using administrative data, we estimated the cost of implementing potential attraction and retention strategies per health worker year worked.

**Results:**

Although health workers preferred urban jobs to rural jobs (OR 1.39, 95% CI 1.11–1.75), employment incentives influenced health workers’ decision to choose rural jobs. If superior housing was offered in a rural area compared to a basic housing allowance in an urban job, participants would be five times as likely to choose the rural job (OR 5.04, 95% CI 4.12–6.18). Education incentives and facility-based improvements also increased the likelihood of rural job uptake. Housing benefits were estimated to have the lowest total costs per health worker year worked, and offer high value in terms of cost per percentage point increase in rural job uptake.

**Conclusions:**

Non-monetary incentives such as housing, education, and facility improvements can be important motivators of health worker choice of location and could mitigate rural health workforce shortages. These results can provide valuable insight into the types of job attributes and incentives that are most likely to be effective in attracting and retaining health workers in rural areas.

**Electronic supplementary material:**

The online version of this article (10.1186/s12960-019-0359-3) contains supplementary material, which is available to authorized users.

## Background

An adequate health workforce is critical to the delivery of health services. With approximately 61% of Zambia’s population living in rural areas [1], ensuring access to services in rural areas is central to achieving national health goals. Zambia suffers from a national human resources for health (HRH) shortage, with 11.2 doctors, nurses, and midwives per 10 000 persons, which is well below the WHO minimum recommended threshold of 22.8 doctors, nurses, and midwives per 10 000 persons [2]. As of 2017, approximately 10% of Zambia’s health workforce was lost each year to attrition [3]. Although there has been an overall increase in the number of clinical health workers since 2008, shortages remain highest in rural areas. While the majority of Zambia’s population live in rural areas, the total number of doctors, nurses, and midwives in rural areas stands at 7677 compared to 9285 in urban areas [2]. An analysis of the workforce in 2016 showed that 45% of doctors, clinical officers, nurses, and midwives were working in rural areas [2].

In 2003, the Ministry of Health (MOH) launched the Zambian Health Workers Retention Scheme (ZHWRS) pilot program to attract and retain medical doctors in rural and remote areas of the country. The scheme was expanded in 2007 to include environmental health technicians, nurses, medical teaching staff, medical consultants, medical licentiates, and clinical officers. The ZHWRS ranks districts by level of remoteness and offers various benefits to health worker in each district, such as hardship allowances, housing rehabilitation funds, vehicle loans, utility equipment (solar panels or a bore hole), radio equipment, and professional development priority [4]. Evaluations of the ZHWRS have suggested that the policy has had limited effectiveness and that none of the included incentives are significantly associated with higher retention in rural areas [5–7]. In response, the MOH developed the ZHWRS Sustainability Strategy (ZHWRS-SS) in 2014. The ZHWRS-SS called for an effectiveness evaluation and cost-benefit analysis of other potential non-monetary incentives in order to inform the selection of sustainable incentives that could replace those included in the ZHWRS.

The aim of this study was to identify policy-applicable strategies that are most likely to attract and retain health workers in rural areas of Zambia, and to examine the relative costs and benefits of the strategies. A multi-country study in South Africa, Kenya, and Thailand found that nurses’ preferences varied significantly across countries and recommended that intervention packages should be tailored to local conditions [8]. So while MOH policymakers in Zambia could gain general information from the global evidence base, data emerging from their specific context was critical. Furthermore, few past studies have rigorously examined the costs of proposed incentives and considered health worker preferences in light of costs per health worker year worked.

This study adopted a discrete choice experiment (DCE) design. In recent years, DCEs have emerged as an important tool for examining the potential effects of HRH interventions by understanding the preferences of health workers and the probability of uptake of defined jobs [9, 10]. Previous research has highlighted several key types of strategies or incentives for attracting and retaining health workers in rural areas [11–13], including educational opportunities, financial incentives, professional support and recognition, improved living conditions and personal transportation, regulatory policy change, and improved facility conditions. The details of these packages are, however, contextually dependent on the status quo, health worker preferences, and political and financial feasibility.

## Methods

This study was approached through two phases: development of a DCE questionnaire based on interviews with health workers, focus group discussions (FGDs) with students, and government consultations, and execution and analysis of a DCE questionnaire to elicit information on preferences for attribute packages from practicing health workers and health professional students.

### Qualitative data collection and questionnaire development

The purpose of the qualitative component was to inform the list of job attributes that would be used to construct the job choice sets included in the DCE [9, 14]. Semi-structured, in-depth interviews were held with 25 health workers employed in rural health centers. FGDs were conducted with students in their last year of training, including 16 doctors, 22 clinical officers, and 215 midwives and nurses. Locations for health worker interviews and training institutions for FGDs were selected to prioritize inclusion of health workers and students with experience in rural areas. In the interviews and FGDs, participants were asked to discuss sources of satisfaction or dissatisfaction in their past or current postings and possible incentives that could be offered to encourage them to take up or stay in postings in rural or remote areas. At the end of each interview or FGD session, participants were asked to review a list of job attributes and identify four that were most important. The qualitative phase of this study is described in detail in a separate technical report (Kamanga, A, et al. Push and Pull factors: Rural Retention of Health Workers in Zambia, manuscript in preparation.).

Responses from the ranking exercise were coded and categorized, and each attribute was given an overall rank based on participant input. Policymakers from the MOH considered the attribute rankings, as well as the feasibility of implementing various incentive strategies aimed at impacting these job attributes, and agreed that the DCE should include the following attributes: (1) salary-based incentives (to enable cost-benefit analysis), (2) educational opportunities, (3) housing (including references to utility status), (4) facility transportation, and (5) medical equipment. Within each attribute category, levels were created based on the qualitative descriptions from participants as well as implementation feasibility. An overview of the attributes and levels is shown in Table 1.

**Table 1:** Job characteristics presented in discrete choice experiment

Attribute	Job attribute levels	Offered in rural job	Offered in urban job
Salary	• Base salary	✓	✓
	• Base salary, plus 20% rural allowance	✓	
	• Base salary, plus 25% rural allowance	✓	
	• Base salary, plus 30% rural allowance	✓	
Educational opportunities	• No scholarship after 2 years: guaranteed paid leave after 2 years with no government financial assistance for study	✓	✓
	• 75% scholarship after 3 years: guaranteed paid leave after 3 years with eligibility for (not guaranteed) 75% government scholarship for study	✓	
	• 100% scholarship after 4 years: guaranteed paid leave after 4 years with 100% government scholarship for study guaranteed	✓	
Housing	• Basic allowance offered (20% of base salary), but no housing provided	✓	✓
	• Basic housing provided (two bedrooms, outside bathroom, no electricity, water available through bore hole or hand pump)	✓	✓
	• Superior housing provided (three bedrooms, electricity, piped running water, self-contained master bedroom and security reinforcements)	✓	✓
	• Superior allowance offered (30% of base salary), but no housing provided	✓	
Transportation	• None: no access to ambulance or utility vehicle	✓	✓
	• Available: reliable access to ambulance and utility vehicle (motorbike/vehicle) for official facility use only	✓	✓
Medical equipment	• Inadequate: standard list of medical equipment at health facility *not* always available	✓	✓
	• Adequate: standard list of medical equipment at health facility always available	✓	✓

A labeled design was used for this DCE, whereby each choice set included one rural and one urban job. The alternative label conveys information to participants that is not explicitly stated in the choice set and therefore provides preference results that are more realistic and less abstract [15]. A labeled design also provides the opportunity to create a restricted design, which limits certain attribute levels to only be available in rural or urban locations. Through a restricted design, the realism of the options provided was enhanced by avoiding implausible combinations (such as the government offering a rural allowance for an urban job).

The selected DCE attributes and levels were matched to create job choices using R software. Given the attributes and levels selected, a total of 2304 pairs of job sets were possible, and a fractional factorial design was used to select a fraction of the total job choice sets to be presented to participants to optimize D-efficiency, maximize level balance and orthogonality, and minimize overlap among attribute levels [9]. The questionnaire was organized into two blocks of 12 choice sets, and participants were randomly assigned to a block. In addition to the DCE choice sets, demographic and background questions were included. The final DCE questionnaire is included as Additional file 1.

### Sampling and data collection

The questionnaire was tested with approximately 20 participants to ensure general comprehension of the instructions and questionnaire design. The anonymous questionnaire was completed by 474 participants between October 2016 and January 2017.

Participants were conveniently and purposefully sampled with the goal of obtaining a sample of participants with balanced representation of key demographic characteristics, and that was generally representative of the Zambian health workforce with regard to the health worker cadres represented. Given the focus on understanding retention of health workers, the sample was also designed to include primarily health workers with rural experience, since it was assumed that these individuals would be able to most realistically assess the value of incentives offered. The sample was recruited through three approaches: (1) Students in their final year of pre-service or up-grading training at five training institutions were invited to participate. (2) Active health workers were sampled from a nationwide series of training workshops for supervisors of community health assistants (CHAs) in five provinces: Luapula, Northern, Western, Northwestern, and Muchinga. Since the CHA cadre exclusively works in rural and remote areas, these workshops included staff currently working and residing in rural areas. (3) Health workers were selected from health facilities in rural districts of two additional provinces (Eastern and Central). Overall, participants were drawn from a total of 26 districts, of which 24 are classified as rural or remote by the Zambian Public Service Management Division (PSMD).

All survey participants provided written consent prior to participation. Data was collected using paper-based questionnaires. Data entry was conducted using EpiData, and 25% of the questionnaires were double entered to ensure accuracy.

### Data analysis

Demographic, education, and work experience characteristics were analyzed using univariate, descriptive statistics. Bivariate logistic regression was used to explore associations between the stated likelihood of working in a rural area in the future and various demographic or background characteristics. A conditional logit model was used to investigate the preferences for job attributes among participants. Data were analyzed as categorical dummy variables that represented the existence of a certain attribute level in either a rural or an urban setting. These dummy variables can be thought of as interaction variables between the attribute level and the location; such an approach is made possible based on the labeled study design. Separate conditional logit models were used to explore differences in preferences among different cadre groups and other sub-groups, though the study was not specifically designed to examine differences in these groups because it is unlikely that the government would offer different incentives to different sub-groups of employees.

An uptake rate or preference impact measure was calculated to estimate the percentage of health workers that would prefer a job posting that offers a specific package of incentives as compared to other job postings [16]. Several validity tests were conducted to determine the appropriateness of model specifications. Specifically, we investigated dominance and internal or predictive validity. Dominance in a DCE means that a participant always selected job scenarios on the basis of one attribute (for example, a participant always chose rural jobs). Such behavior challenges the basic assumption of random utility theory that individuals make trade-offs between various characteristics in making choices [17]. In order to assess internal or predictive validity, we used the uptake prediction values for actual scenarios presented in the questionnaire to understand whether our predicted uptake for a job scenario matched the percentage of participants that actually chose that job scenario [18, 19]. All analyses were performed using Stata 13.

## Results

Data was collected from 474 participants, including 259 (54.6%) active health workers (sampled from health facilities and regional in-service training) and 215 (45.4%) students (sampled from five training institutions). Table 2 presents the demographic characteristics of the participants. The sample is balanced across key demographic characteristics, and cadres are represented in a way that is roughly proportionate to their size within the national health workforce (see table notes).

**Table 2:** Sample demographic characteristics

Characteristic	*n* (%)^1^
Gender	
Female	242 (51.1)
Male	231 (48.7)
Age	
19 years or younger	1 (0.2)
20 to 29 years	289 (61.0)
30 to 39 years	115 (24.3)
40 to 49 years	36 (7.6)
50 years or older	30 (6.3)
Marital status	
Single	226 (47.7)
Engaged	57 (12.0)
Married	178 (37.6)
Widowed or divorced	9 (1.9)
Dependents	
Has dependents	282 (59.5)
No dependents	185 (39.0)
Health worker status	
Active health worker	259 (54.6)
Student	215 (45.4)
Years practicing as a health worker	
No health worker experience	92 (19.4)
Less than 1 year	135 (28.5)
1 to 3 years	73 (15.4)
4 to 6 years	55 (11.6)
7 to 10 years	28 (5.9)
More than 10 years	78 (16.5)
Experience as health worker in rural areas	
None or less than 1 year	243 (51.3)
1 year or more	215 (45.4)
Health worker cadre^2^	
Enrolled nurse	82 (17.3)
Registered nurse	176 (37.1)
Enrolled midwife	39 (8.2)
Registered midwife	11 (2.3)
Environmental health officer	90 (19.0)
Clinical officer	41 (8.7)
Medical doctor	18 (3.8)
Stated likelihood of working in a rural area in the future	
Very likely	113 (23.8)
Likely	235 (50.0)
Unlikely	70 (14.8)
Very unlikely	43 (9.1)

^1^Percentages may not sum to 100% due to missing data and rounding

^2^As of a 2016 analysis, the MOH reports that the actual workforce included the following percentages of these cadres (EHTs were not included): nurses (66.3%), midwives (18.9%), clinical officers (10.4%), and doctors (4.4%) [2]

### Reported likelihood of working in a rural area

As shown in Table 2, over 73% of participants reported that they were likely or very likely to work in a rural area in the future. We explored the association between self-reported likelihood of working in a rural area and participant characteristics (Table 3). Participants were more likely to report likelihood of working in a rural area in the future if they had limited work experience (odds ratio (OR) 1.67, *p* value 0.02) or had limited rural work experience (OR 1.53, *p* value 0.05). Students were twice as likely to say they would take up a rural position compared to active health care workers (OR 2.04, *p* value < 0.01). Finally, participants who rated their past work experience in rural areas as excellent had 12 times higher odds of indicating likelihood of taking up a rural position (OR 12.38, *p* value < 0.01), and those who rated their experience as good had nearly six times higher odds (OR 5.90, *p* value < 0.01). Those who rated their past experience working in a rural area as fair had 3.85 times higher odds (3.85, *p* value < 0.01).

**Table 3:** Association between demographics and experiences with stated likelihood of working in a rural area in the future

Characteristic	Likely or very likely to work in rural area *n* (%)^1^	Unlikely or very unlikely to work in rural area *n* (%)^1^	Odds ratio (*p* value)^2^
Gender			
Female	167 (73.6)	60 (26.4)	1.22 (0.36)
Male	180 (77.3)	53 (22.8)	*ref.*
Age			
29 years or younger	212 (76.3)	66 (23.7)	1.14 (0.57)
30 years or older	133 (73.9)	47 (26.1)	*ref.*
Marital status			
Single or divorced	183 (80.3)	45 (19.7)	1.65 (0.02)
Married or engaged	163 (71.2)	66 (28.8)	*ref.*
Dependents			
No children	139 (78.5)	38 (21.5)	1.31 (0.24)
Has children	204 (73.7)	73 (26.4)	*ref.*
Health worker status			
Student	170 (82.5)	36 (17.5)	2.04 (< 0.01)
Active health worker	178 (69.8)	77 (30.2)	*ref.*
Years practicing as a health worker			
None or less than 1 year	178 (80.2)	44 (19.8)	1.67 (0.02)
More than 1 year	165 (70.8)	68 (29.2)	*ref.*
Years practicing as a health worker in rural areas			
None or less than 1 year	188 (79.0)	50 (21.0)	1.53 (0.05)
More than 1 year	152 (71.0)	62 (29.0)	*ref.*
Bonding agreement or other rural obligation			
Has bonding agreement or other obligation	56 (84.9)	10 (15.2)	2.04 (0.05)
No bonding agreement or other obligation	280 (73.3)	102 (26.7)	*ref.*
Rating of experience working in rural area			
Excellent	55 (90.2)	6 (9.8)	12.38 (< 0.01)
Good	118 (81.4)	27 (18.6)	5.90 (< 0.01)
Fair	117 (74.1)	41 (26.0)	3.85 (< 0.01)
Poor	20 (42.6)	27 (57.5)	*ref.*
Health worker cadre			
Environmental health officer	52 (58.4)	37 (41.6)	*ref.*
Nurse	198 (79.2)	52 (20.8)	2.71 (< 0.01)
Midwife	41 (83.7)	2 (16.3)	3.64 (< 0.01)
Clinical officer	35 (85.4)	6 (14.6)	4.15 (< 0.01)
Medical doctor	12 (66.7)	6 (33.3)	1.42 (0.52)

^1^Percentages are row percentages, but may not sum to 100% due to missing data. OR denotes “odds ratios” which represents the odds that an outcome will occur given a certain exposure (or in this characteristic)

^2^*p* values and odds ratios are based on bivariate logistic regressions

### Job attribute preferences

In total, 78 participants (16.5%) expressed a dominant preference for a certain job characteristic (rural job, 62 participants; urban job, 15 participants; education after 2 years with no scholarship, one respondent). Secondary analysis demonstrated that this is likely to represent true opinions of this group rather than a problem with survey comprehension because there was internal consistency between the job set responses of these participants and the responses they gave in the introductory section of the questionnaire about likelihood of working in rural areas.

The results from the conditional logit model showed that superior housing had the strongest influence on rural job choice (Table 4). Specifically, participants were 5.04 times more likely to select the rural job if superior housing was offered compared to a basic housing allowance (95% CI 4.12–6.18, *p* value < 0.01). The second most influential characteristic was a 100% scholarship to return to school for upgrading after 4 years of service. Participants were nearly twice as likely to take this option compared to a job with no scholarship but education leave after 2 years of services (OR 1.98, 95% CI 1.69–2.33, *p* value < 0.01). Superior housing was also offered in urban locations and was influential in those settings but to a lesser degree compared to rural settings (OR 2.21, 95% CI 1.86–2.62, *p* value < 0.01). In separate models, assessing responses across cadres and within sub-groups based on the level of rural work experience of participants (1 year or more versus less than 1 year), results were similar with no significant differences between groups.

**Table 4:** Determinants of job preferences for all cadres

Incentive category and level	Odds ratio	95% CI	*p* value
Location			
Rural (ref)	–	–	–
Urban	1.39	1.11–1.75	0.01
Rural job characteristics			
Salary			
Base salary (ref)	–	–	–
Base salary + 20% rural allowance	1.19	1.00–1.43	0.05
Base salary + 25% rural allowance	1.41	1.15–1.73	< 0.01
Base salary + 30% rural allowance	1.52	1.25–1.86	< 0.01
Educational opportunities			
0% scholarship after 2 years (ref)	–	–	–
75% scholarship after 3 years	1.25	1.06–1.48	0.01
100% scholarship after 4 years	1.98	1.69–2.33	< 0.01
Housing			
Basic allowance (ref)	–	–	–
Basic housing	1.12	0.91–1.37	0.29
Superior housing	5.04	4.12–6.18	< 0.01
Superior allowance	1.44	1.22–1.70	< 0.01
Transportation			
None (ref)	–	–	–
Available	1.45	1.27–1.65	< 0.01
Medical equipment			
Inadequate (ref)	–	–	–
Adequate	1.10	0.96–1.26	0.18
Urban job characteristics			
Housing			
Basic allowance (ref)	–	–	–
Basic housing	0.88	0.73–1.05	0.17
Superior housing	2.21	1.86–2.62	< 0.01
Transportation			
None (ref)	–	–	–
Available	1.31	1.15–1.50	< 0.01
Medical equipment			
Inadequate (ref)	–	–	–
Adequate	1.39	1.21–1.59	< 0.01
Model diagnostics			
Number of participants	474		
Number of observations	9 316		
Log likelihood	−2 793.05		
Pseudo *R*^2^	0.1349		
Prob > chi-square	< 0.001		

### Predicted job uptake

**Fig. 1:**
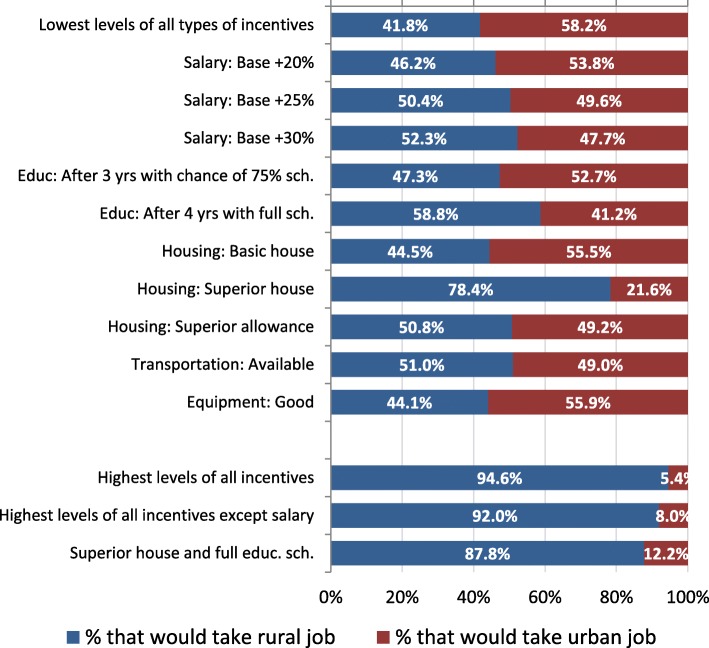
Expected rural and urban job choice given various job characteristics. This figure assesses the impact of offering superior incentives in the rural job compared to the base level of each incentive category in an urban job

Using the results from the conditional logit model, we transformed the data to estimate the percentage of health workers that would choose the rural or urban job if specific attraction and retention strategies were implemented (Fig. 1). With the base or reference level of job characteristics in both the rural and urban job, 41.8% of health workers would take the rural job and 58.2% would take the urban job. If superior housing was offered in a rural job compared to the basic housing allowance in an urban job, 78.4% of health workers would be expected to take the rural job. In other words, an additional 36.6% of health workers would be convinced to take a rural job based on the provision of superior housing.

However, in practice, the choice that many health workers face is not between urban and rural job with the base level of all incentives. Instead, many health workers choose between a rural job with a 20% rural allowance and basic incentives compared to an urban job with access to transportation and good quality medical equipment, but basic job characteristics on all other counts. In this scenario, our model predicts that 32.0% of health workers would take the rural job under these circumstances.

### Cost of job attributes

Understanding the costs of these incentives relative to their potential benefits is critical for policymakers. Costs were estimated using the salary and upgrading requirements of registered nurses and are calculated. Since attribute preferences are presented based on marginal benefits of one level over another, costs are calculated as marginal costs. Additional details about the assumptions made in costing these incentives are included in Additional file 2.

Overall, the housing options have the lowest total costs per working year (ranging from US$932 to US$1398), since houses can retain health care workers over a 25-year period despite high initial construction costs and continued maintenance costs. Individual education incentives have the largest total costs, with the main driver of costs being the salary paid while the health worker is on study leave. However, when considering the marginal costs of the higher-level incentives compared to the base, the marginal costs of the education incentives are low since delaying the return to school by 3 or 4 years (rather than 2) offsets the cost of the scholarship. In fact, one of the education incentives has a lower total cost than the base level leading to a negative marginal cost. By dividing the marginal costs of each job attribute by the estimated increase in rural job uptake (Fig. 1), estimates were developed for the cost per percentage point increase in rural job uptake (Table 5). This analysis shows that the cost of the education options per percentage point increase is low due to their low marginal costs. However, even though a chance of a 75% scholarship after 3 years is cheaper than the base level, it was not found to be a strong predictor of rural job uptake (Table 4). Aside from education, the options that offer the most benefit a lowest cost are in the housing category. Specifically, superior housing allowance offers an increase of 9.0 percentage points at a cost of $52 per percentage point increase, and superior housing offers an increase of 36.6 percentage points at a cost of $49 per percentage point increase.

**Table 5:** Cost per percentage point increase in rural job uptake

		Total cost per working year (USD)	Marginal cost per working year compared to base level (USD)^1^	Percentage of participants that would choose rural job	Percentage point increase in rural uptake^2^	USD per percentage point increase^3^
Salary						
*Ref*	Base salary^4^	$7084	–			
1	Base salary + 20% rural allowance	$ 8016	$932	46.2%	4.4	$214
2	Base salary + 25% rural allowance	$8249	$1 165	50.4%	8.6	$136
3	Base salary + 30% rural allowance	$8482	$1398	52.3%	10.5	$134
Housing						
*Ref*	Basic allowance offered (20% of base salary)	$932	–			
4	Basic housing provided	$1732	$800	44.5%	2.7	$297
5	Superior allowance offered (30% of base salary)	$2732	$466	50.8%	9.0	$52
6	Superior housing provided	$1398	$1800	78.4%	36.6	$49
Education						
*Ref*	Guaranteed paid leave after 2 years with no government financial assistance for study	$9088	–			
7	Guaranteed paid leave after 3 years with eligibility for (not guaranteed) 75% government scholarship for study	$8932	− $156	47.3%	5.5	− $28
8	Guaranteed paid leave after 4 years with 100% government scholarship for study guaranteed	$9332	$244	58.8%	17.0	$14
Transport						
*Ref*	No access to ambulance or utility vehicle	–	–			
9	Reliable access to ambulance and utility vehicle (motorbike/vehicle) for official facility use only	$4250	$4250	51.0%	9.2	$462
Medical equipment						
*Ref*	Standard list of medical equipment at health facility *not* always available	–	–			
10	Standard list of medical equipment at health facility always available	$3167	$3167	44.1%	2.3	$1377
Attribute combinations^5^						
Highest levels of all (3, 6, 8, 9, 10)		–	$7692	94.6%	52.8	$146
Highest levels of all except salary (6, 8, 9, 10)		–	$6294	92.0%	50.2	$125
Superior housing (6) + full scholarship after 4 years (8)		–	$2044	87.8%	46.0	$44
Superior housing allowance (5) + full scholarship after 4 years (8)		–	$710	67.2%	25.4	$28

^1^This table presents the marginal cost or difference in the cost of each higher-level job attribute compared to its reference, because this approach to costing aligns with the odds ratio and percentage uptake results, which compare to marginal benefit of each higher-level job attribute relative to uptake given the reference category. For example, in the case of salary, the reference attribute costs $7084 and salary plus a 20% rural allowance costs $8016, so the marginal cost of salary plus a 20% rural allowance is $932

^2^The percentage point increase in rural job uptake is based on a baseline rural uptake rate of 42.8% before the addition of any higher-level job attributes

^3^The cost (in USD) per percentage point increase provides a standardized way of looking at the cost benefit of each attribute. This value is the marginal cost of the job attribute compared to the reference divided by the marginal benefit in uptake of the attribute compared to the reference. For example, the marginal cost of salary plus a 20% rural allowance is $932, which is divided by 4.4 percentage points to get a cost per percentage point increase in uptake of $214

^4^The salary assumptions are based on the MOH policies for salary of a registered nurse as of 2017. Values would vary slightly for other cadres, but registered nurses were the focus of this analysis because they make up a large portion of the workforce and because of the government interest in retaining this cadre in particular in rural areas. The value is inclusive of standard benefits such as for housing and transport, as described in Additional file 2, but the 20% allowance is calculated using the base salary only, prior to allowances

^5^The numbers in this section refer to the job attributes as numbered in the top part of the table. Each attribute combination is compared to the reference category for all relevant job attributes

## Discussion

The results of this study indicate that superior housing and education incentives in the form of a full scholarship for education are most likely to influence health worker job preferences. Additionally, improvements to facility quality and working environment can support health worker attraction and retention in rural areas. Against the backdrop of Zambia’s rural health workforce shortages, our findings provide evidence to enable retention policies to be shaped around health worker preference. Indeed, these results were considered for and incorporated into the prioritization of retention approaches in the 2018–2024 Zambia Human Resources for Health Strategic Plan [3].

While many DCEs have included some form of housing incentive, a limited number of DCEs have explored superior housing as an offering in comparison to basic housing. These studies did not find superior housing to be a highly influential attribute, though the definition of superior housing varies across studies [8, 20]. Still, in Zambia, superior housing was found to be extremely influential in health worker choices, with an effect estimate that was substantially higher than that of any other attribute, possibly pointing to differences in the local context and typical conditions confronted by health workers in Zambia. As with all potential strategies, the impact should be measured against the costs. When costs are considered on a per health worker year basis and relative to the percentage point increase in job uptake, housing is a cost-effective investment.

This study explored several dimensions of education incentives, including whether a scholarship was offered at all, whether the scholarship was guaranteed, and service time before receiving study leave. These results indicated that health workers are willing to delay study leave if some level of scholarship is offered and have a preference for waiting longer if the scholarship is guaranteed. The costing component of this study showed that the most significant costs associated with study leave are those required to pay salary and basic allowance while health workers are in school, rather than the scholarship itself. When study leave is delayed and those salary costs are distributed over a larger number of working years, costs are offset. As a result, delayed education incentives may offer substantial benefit at relatively low cost. When assessing the appropriateness and feasibility of educational incentives, it is also important to consider the demand for health workers with various levels of credentials. Based on the preferences of health workers, the cost of education, and the skills mix needed in Zambia, education incentives should be considered for a limited number of hard-to-fill positions, but should not be offered widely to all health workers in rural areas.

Enhancements to the facility can lead to important improvements in the quality of services offered to patients. Facility conditions are basic in most rural facilities in Zambia, so the base attribute level was designed to reflect this current reality, with higher-level attributes representing potential investments that could be made to enhance facility quality. Taken individually, facility-based improvements in working environment for health workers, in the form of transport for facility use or improved medical equipment, had limited impact on the job preferences of health workers in this study. However, other research suggests that a bundle of improvements to address multiple needs of health workers, including housing, educational opportunities, and improved working environment, are most likely to be effective in retaining health workers in the long term [21]. This study found that implementing multiple types of facility improvements together could have a larger influence on health worker preferences, and this impact can be increased by including housing and/or education incentives. The idea of facility bundles is in line with the policy direction of the ZHWRS Sustainability Strategy (ZHWRS-SS), which recommended investing in facility-based incentives over individual incentives. Beyond potential health worker recruitment and retention benefits, facility-level improvements are likely to have important and direct impacts on the accessibility and quality of patient care, but these benefits are not measured in this study. Although many health worker retention schemes internationally, including the ZHWRS in Zambia, have focused on the provision of financial incentives for rural service, results from this study indicate that non-monetary incentives such as education and housing benefits have more impact on health worker preferences than rural salary allowances. This is consistent with the results from other research, which indicates that increased salaries alone are not necessarily a sufficient tool for addressing poor retention and health worker motivation. For example, two studies in South Africa have demonstrated that higher salaries alone were not cost-effective in attracting health professionals to rural areas [22, 23] and other DCE studies have indicated that salary was not the most influential of job attributes [18]. Another analysis using data from multiple African countries found that wage differentials were not a major factor influencing health worker migration [24].

Although this study can offer valuable insights into the job preferences of health workers, several limitations should be considered. First, no questionnaire, including a DCE, can capture the full complexity of real-life choices. For example, reference to a “rural” job may invoke a range of different assumptions for various individuals, and other characteristics that are not addressed in this questionnaire, such as language or distance to one’s family home, may play a role in decision. Second, the sample for this DCE was selected using both convenient and purposeful sampling strategies and may not be representative of the national workforce in some ways. The sampling strategy aimed to and provided a sample that reflects the national workforce in several key demographic ways, such as in the cadre breakdown, and includes health workers from a wide range of geographic regions. Third, the DCE questionnaire included 10 possible incentives which were deemed administratively and financially feasible by policymakers in Zambia, but it is possible that other incentives or job characteristics not included here could have influence on health worker job preference. However, the purpose of the qualitative assessment and stakeholder consultations was to ensure that the DCE included the incentives most likely to be influential and feasible to implement. Fourth, this study was meant to examine the role of specific job characteristics in attracting and retaining health workers in rural service, but we are not able to differentiate the impact of job attributes at various time points in a health worker’s career. From a policy standpoint, the government is unlikely to offer different incentives to existing and new employees, so we opted for an approach that included current health workers and new graduates in the sample and where the survey was framed such that participants were asked to consider which job they would be more likely to choose and stay in. Finally, 16% of the participants in this study expressed a dominant preference for jobs with one characteristic. While the responses of these individuals were internally consistent with their responses to background questions, it was necessary to remove these individuals from the analysis because their responses violate model assumptions. Accordingly, our results represent the preferences of the 84% of participants that were willing to trade off between attributes or incentives. The preferences of other 16% of the participants are unlikely to be influenced by incentives provided.

This study focused on strategies that were feasible to be offered by the MOH. However, many factors well beyond the scope of the MOH will influence the quality of life of that health worker and his or her family, such as the job opportunities for spouses, reliability of public utilities, commercial life of the area, quality of the schools for dependents, and the quality of road networks. Therefore, rural development will require an effort from many governmental sectors. The MOH should explore opportunities to partner with other ministries that may be experiencing similar rural retention challenges, such as the Ministry of Education, in order to identify areas for collaboration.

## Conclusion

There is an urgent need for new strategies to improve rural health worker attraction and retention in rural Zambia to address Zambia’s rural health workforce shortages. This study provides valuable evidence on the policy interventions that are most likely to be effective, relative to the cost of the investment. It builds on previous research which found that the salary incentives offered under the previous ZHWRS were not effective in improving health workers’ job satisfaction, likelihood of leaving, or frequency of considering leaving [7]. This study investigated a different set of interventions, developed and prioritized through qualitative research with health workers and students. The results indicate that housing incentives and educational scholarships for rural health workers are likely to be most effective in strengthening the rural health workforce, and based on an analysis of the costs of these incentives, housing incentives offer the best value for money. This study provides policymakers in Zambia with practical information that can be used to inform decisions about how to invest in strengthening the rural health workforce. Beyond Zambia, these results can inform policy discussions, though context-specific information is likely to be needed in other countries. Our approach to costing of incentives represents a unique view not taken in other published literature and could inform the methodology for studies in other locations.

## Additional files

Additional file 1: Discrete choice experiment questionnaires. (DOCX 64 kb)

Additional file 2: Costing detail. (DOCX 37 kb)

## Abbreviations

CHA: Community Health Assistant
CHAI: Clinton Health Access Initiative, Inc.
DCE: Discrete choice experiment
FGD: Focus group discussion
MOH: Ministry of Health
PSMD: Public Service Management Division
USD: US Dollar
ZHWRS: Zambian Health Workers Retention Scheme
ZMW: Zambian Kwacha
