# Supplementary material for: Assessment of interventions to attract and retain health workers in rural Zambia: a discrete choice experiment
Source: Hum Resour Health. 2019 Apr 3;17:26. doi: 10.1186/s12960-019-0359-3 (PMC6448309; doi:10.1186/s12960-019-0359-3)
Supplement: Supplementary file 1 — Discrete choice experiment questionnaires. (DOCX 64 kb) [file 12960_2019_359_MOESM1_ESM.docx]

***Additional file 1: Discrete choice experiment questionnaires***

*Notes: This file contains two questionnaires – Block 1 and Block 2. Each block includes different job choice sets, and respondents were randomly assigned to receive one of the two questionnaires.*

**BLOCK 1**

| **PART 1. DEMOGRAPHIC QUESTIONS** | |
| --- | --- |
| *Instructions: Please answer the following questions to the best of your ability.* | |
| 1. **What is your gender?** | 🞏 Male  🞏 Female |
| 1. **What is your age group?** | 🞏 19 years or younger  🞏 20 to 29 years  🞏 30 to 39 years  🞏 40 to 49 years  🞏 50 years or older |
| 1. **What is your marital status?** | 🞏 Single  🞏 Engaged  🞏 Married  🞏 Divorced or widowed |
| 1. **Do you have any dependents that live with you?** | 🞏 Yes  🞏 No |
| 1. **Please indicate which age group(s) your children fall into. (Check all that apply)** | 🞏 Under 2 years  🞏 2 to 6 years  🞏 7 to 12 years  🞏 12 to 18 years  🞏 Older than 18 years  🞏 Not applicable |
| 1. **Have you at any time lived in a rural area for more than 1 year at a time?** | 🞏 Yes  🞏 No |

| **PART 2. PROFESSIONAL BACKGROUND QUESTIONS** | |
| --- | --- |
| *Instructions: Please answer the following questions to the best of your ability.* | |
| 1. **Are you currently a Student or a Health Care Worker** | 🞏 Health Care Worker  🞏 Student (Pre-service)  🞏 Student (In-service) |
| 1. **Indicate your professional cadre** | 🞏 Enrolled Nurse  🞏 Registered Nurse  🞏 Enrolled Midwife  🞏 Registered Midwife  🞏 Environmental Health Technologist  🞏 Clinical Officer  🞏 Medical Licentiate  🞏 Medical Doctor  🞏 Other __________________ |
| 1. **Has your experience been in the public or the private sector?** | 🞏 All public sector  🞏 Both public and private sector  🞏 All private sector |
| 1. **How many years have you worked as a health worker?** | 🞏 Less than 1 year  🞏 1 to 3 years  🞏 4 to 6 years  🞏 7 to 10 years  🞏 11 or more years  🞏 Not applicable |
| 1. **How many years have you worked as a health worker in rural areas?** | 🞏 Less than 1 year  🞏 1 to 3 years  🞏 4 to 6 years  🞏 7 to 10 years  🞏 11 or more years |
| 1. **Please rate your overall experience working in rural areas?** | 🞏 Excellent or very good  🞏 Good  🞏 Fair  🞏 Poor  🞏 Fair  🞏 I have never worked in a rural area |
| 1. **Are you under any obligation or have you made any commitment to work in a rural area for a certain amount of time or in exchange for some benefit?** | 🞏 Yes  🞏 No |
| 1. **Are you planning to return to school for any type of specialty or upgraded training?** | 🞏 Yes, within the next two years  🞏 Yes, but not until after working for two or more years  🞏 No |
| 1. **Please rate how likely you are to work in a rural area in the next 5 years.** | 🞏 Very likely  🞏 Likely  🞏 Unlikely  🞏 Very unlikely |

| **PART 3. JOB POSTING CHOICE SETS** |
| --- |
| *Instructions:*   - *Imagine that you have applied for a job or transfer of posting, and you have been given options about which job you would prefer to take.* - *In each of the following choice sets, please indicate whether you would be more likely to accept Job Posting A or Job Posting B, based on the information provided.* - *Job A is always in a rural location and Job B always is in an urban location.* |

**An example of basic salary with percentage allowances**

| **Basic salary (per month)** | **20% allowance** | **25% allowance** | **30% allowance** |
| --- | --- | --- | --- |
| **K2,800** | **K560** | **K700** | **K840** |
| **K3,200** | **K640** | **K800** | **K960** |
| **K3,500** | **K700** | **K875** | **K1,050** |
| **K7,376** | **K1,475** | **K1,844** | **K2,213** |

| **JOB POSTING CHOICE SET #1** | | |
| --- | --- | --- |
|  | **Posting A** | **Posting B** |
| ***Location*** | Rural | Urban |
| ***Salary*** | Basic salary + 20% rural allowance | Basic salary |
| ***Educational opportunities*** | After 2 years:   - Guaranteed paid study leave - NO government sponsorship provided | After 2 years:   - Guaranteed paid study leave - NO government sponsorship provided |
| ***Housing*** | BASIC HOUSING:   - 2 bedrooms - Outside bathroom - No electricity - Water available through bore hole or hand pump | BASIC ALLOWANCE:   - No housing offered - 20% of Basic salary housing allowance provided |
| ***Transport*** | NONE: No access to ambulance no utility vehicle | NONE: No access to ambulance no utility vehicle |
| ***Facility equipment*** | INADEQUATE: Standard list of medical equipment at health facility NOT always available | INADEQUATE: Standard list of medical equipment at health facility NOT always available |
| **Which posting would you choose?** | 🞏 | 🞏 |

| **JOB POSTING CHOICE SET #2** | | |
| --- | --- | --- |
|  | **Posting A** | **Posting B** |
| ***Location*** | Rural | Urban |
| ***Salary*** | Basic salary | Basic salary |
| ***Educational opportunities*** | After 3 years:   - Guaranteed paid study leave - Eligible (not guaranteed) for 75% government sponsorship | After 2 years:   - Guaranteed paid study leave - NO government sponsorship provided |
| ***Housing*** | SUPERIOR HOUSING:   - 3 bedrooms - Electricity - Piped running water - Self-contained master bedroom - Security reinforcements (fence, grills, bars on windows) | BASIC ALLOWANCE:   - No housing offered - 20% of Basic salary housing allowance provided |
| ***Transport*** | AVAILABLE: Reliable access to ambulance and utility vehicle (motorbike/ vehicle) for official facility use only | NONE: No access to ambulance no utility vehicle |
| ***Facility equipment*** | INADEQUATE: Standard list of medical equipment at health facility NOT always available | INADEQUATE: Standard list of medical equipment at health facility NOT always available |
| **Which posting would you choose?** | 🞏 | 🞏 |

| **JOB POSTING CHOICE SET #3** | | |
| --- | --- | --- |
|  | **Posting A** | **Posting B** |
| ***Location*** | Rural | Urban |
| ***Salary*** | Basic salary + 30% rural allowance | Basic salary |
| ***Educational opportunities*** | After 3 years:   - Guaranteed paid study leave - Eligible (not guaranteed) for 75% sponsorship | After 2 years:   - Guaranteed paid study leave - NO government sponsorship provided |
| ***Housing*** | BASIC HOUSING:   - 2 bedrooms - Outside bathroom - No electricity - Water available through bore hole or hand pump | SUPERIOR HOUSING:   - 3 bedrooms - Electricity - Piped running water - Self-contained master bedroom - Security reinforcements (fence, grills, bars on windows) |
| ***Transport*** | AVAILABLE: Reliable access to ambulance and utility vehicle (motorbike/ vehicle) for official facility use only | NONE: No access to ambulance no utility vehicle |
| ***Facility equipment*** | ADEQUATE: Standard list of medical equipment at health facility always available | ADEQUATE: Standard list of medical equipment at health facility always available |
| **Which posting would you choose?** | 🞏 | 🞏 |

| **JOB POSTING CHOICE SET #4** | | |
| --- | --- | --- |
|  | **Posting A** | **Posting B** |
| ***Location*** | Rural | Urban |
| ***Salary*** | Basic salary + 20% rural allowance | Basic salary |
| ***Educational opportunities*** | After 3 years:   - Guaranteed paid study leave - Eligible (not guaranteed) for 75% sponsorship | After 2 years:   - Guaranteed paid study leave - NO government sponsorship provided |
| ***Housing*** | BASIC ALLOWANCE:   - No housing offered - 20% of Basic salary housing allowance provided | BASIC HOUSING:   - 2 bedrooms - Outside bathroom - No electricity - Water available through bore hole or hand pump |
| ***Transport*** | AVAILABLE: Reliable access to ambulance and utility vehicle (motorbike/ vehicle) for official facility use only | AVAILABLE: Reliable access to ambulance and utility vehicle (motorbike/ vehicle) for official facility use only |
| ***Facility equipment*** | INADEQUATE: Standard list of medical equipment at health facility NOT always available | ADEQUATE: Standard list of medical equipment at health facility always available |
| **Which posting would you choose?** | 🞏 | 🞏 |

| **JOB POSTING CHOICE SET #5** | | |
| --- | --- | --- |
|  | **Posting A** | **Posting B** |
| ***Location*** | Rural | Urban |
| ***Salary*** | Basic salary + 30% rural allowance | Basic salary |
| ***Educational opportunities*** | After 2 years:   - Guaranteed paid study leave - NO government sponsorship provided | After 2 years:   - Guaranteed paid study leave - NO government sponsorship provided |
| ***Housing*** | SUPERIOR HOUSING:   - 3 bedrooms - Electricity - Piped running water, - Self-contained master bedroom - Security reinforcements (fence, grills, bars on windows) | BASIC HOUSING:   - 2 bedrooms - Outside bathroom - No electricity - Water available through bore hole or hand pump |
| ***Transport*** | NONE: No access to ambulance no utility vehicle | AVAILABLE: Reliable access to ambulance and utility vehicle (motorbike/ vehicle) for official facility use only |
| ***Facility equipment*** | ADEQUATE: Standard list of medical equipment at health facility always available | INADEQUATE: Standard list of medical equipment at health facility NOT always available |
| **Which posting would you choose?** | 🞏 | 🞏 |

| **JOB POSTING CHOICE SET #6** | | |
| --- | --- | --- |
|  | **Posting A** | **Posting B** |
| ***Location*** | Rural | Urban |
| ***Salary*** | Basic salary + 20% rural allowance | Basic salary |
| ***Educational opportunities*** | After 4 years:   - Paid study leave guaranteed - 100% government sponsorship guaranteed | After 2 years:   - Guaranteed paid study leave - NO government sponsorship provided |
| ***Housing*** | SUPERIOR ALLOWANCE:   - No housing offered, - 30% of basic salary housing allowance provided | BASIC HOUSING:   - 2 bedrooms - Outside bathroom - No electricity - Water available through bore hole or hand pump |
| ***Transport*** | AVAILABLE: Reliable access to ambulance and utility vehicle (motorbike/ vehicle) for official facility use only | NONE: No access to ambulance no utility vehicle |
| ***Facility equipment*** | ADEQUATE: Standard list of medical equipment at health facility always available | ADEQUATE: Standard list of medical equipment at health facility always available |
| **Which posting would you choose?** | 🞏 | 🞏 |
| **JOB POSTING CHOICE SET #7** | | |
|  | **Posting A** | **Posting B** |
| ***Location*** | Rural | Urban |
| ***Salary*** | Basic salary + 25% rural allowance | Basic salary |
| ***Educational opportunities*** | After 2 years:   - Guaranteed paid study leave - NO government sponsorship provided | After 2 years:   - Guaranteed paid study leave - NO government sponsorship provided |
| ***Housing*** | SUPERIOR ALLOWANCE:   - No housing offered, - 30% of basic salary housing allowance provided | BASIC HOUSING:   - 2 bedrooms - Outside bathroom - No electricity - Water available through bore hole or hand pump |
| ***Transport*** | AVAILABLE: Reliable access to ambulance and utility vehicle (motorbike/ vehicle) for official facility use only | NONE: No access to ambulance no utility vehicle |
| ***Facility equipment*** | INADEQUATE: Standard list of medical equipment at health facility NOT always available | ADEQUATE: Standard list of medical equipment at health facility always available |
| **Which posting would you choose?** | 🞏 | 🞏 |

| **JOB POSTING CHOICE SET #8** | | |
| --- | --- | --- |
|  | **Posting A** | **Posting B** |
| ***Location*** | Rural | Urban |
| ***Salary*** | Basic salary + 25% rural allowance | Basic salary |
| ***Educational opportunities*** | After 3 years:   - Guaranteed paid study leave - Eligible (not guaranteed) for 75% government sponsorship for a study program | After 2 years:   - Guaranteed paid study leave - NO government sponsorship provided |
| ***Housing*** | SUPERIOR HOUSING:   - 3 bedrooms - Electricity - Piped running water - Self-contained master bedroom - Security reinforcements (fence, grills, bars on windows) | BASIC HOUSING:   - 2 bedrooms - Outside bathroom - No electricity - Water available through bore hole or hand pump |
| ***Transport*** | AVAILABLE: Reliable access to ambulance and utility vehicle (motorbike/ vehicle) for official facility use only | NONE: No access to ambulance no utility vehicle |
| ***Facility equipment*** | ADEQUATE: Standard list of medical equipment at health facility always available | INADEQUATE: Standard list of medical equipment at health facility NOT always available |
| **Which posting would you choose?** | 🞏 | 🞏 |

| **JOB POSTING CHOICE SET #9** | | |
| --- | --- | --- |
|  | **Posting A** | **Posting B** |
| ***Location*** | Rural | Urban |
| ***Salary*** | Basic salary + 30% rural allowance | Basic salary |
| ***Educational opportunities*** | After 3 years:   - Guaranteed paid study leave - Eligible (not guaranteed) for 75% government sponsorship for a study program | After 2 years:   - Guaranteed paid study leave - NO government sponsorship provided |
| ***Housing*** | SUPERIOR ALLOWANCE:   - No housing offered - 30% of basic salary housing allowance provided | SUPERIOR HOUSING:   - 3 bedrooms - Electricity - Piped running water - Self-contained master bedroom - Security reinforcements (fence, grills, bars on windows) |
| ***Transport*** | NONE: No access to ambulance no utility vehicle | NONE: No access to ambulance no utility vehicle |
| ***Facility equipment*** | INADEQUATE: Standard list of medical equipment at health facility NOT always available | INADEQUATE: Standard list of medical equipment at health facility NOT always available |
| **Which posting would you choose?** | 🞏 | 🞏 |

| **JOB POSTING CHOICE SET #10** | | |
| --- | --- | --- |
|  | **Posting A** | **Posting B** |
| ***Location*** | Rural | Urban |
| ***Salary*** | Basic salary | Basic salary |
| ***Educational opportunities*** | After 3 years:   - Guaranteed paid study leave - Eligible (not guaranteed) for 75% government sponsorship for a study program | After 2 years:   - Guaranteed paid study leave - NO government sponsorship provided |
| ***Housing*** | SUPERIOR ALLOWANCE:   - No housing offered - 30% of basic salary housing allowance provided | BASIC ALLOWANCE:   - No housing offered - 20% of Basic salary housing allowance provided |
| ***Transport*** | NONE: No access to ambulance no utility vehicle | AVAILABLE: Reliable access to ambulance and utility vehicle (motorbike/ vehicle) for official facility use only |
| ***Facility equipment*** | ADEQUATE: Standard list of medical equipment at health facility always available | INADEQUATE: Standard list of medical equipment at health facility NOT always available |
| **Which posting would you choose?** | 🞏 | 🞏 |

| **JOB POSTING CHOICE SET #11** | | |
| --- | --- | --- |
|  | **Posting A** | **Posting B** |
| ***Location*** | Rural | Urban |
| ***Salary*** | Basic salary + 30% rural allowance | Basic salary |
| ***Educational opportunities*** | After 2 years:   - Guaranteed paid study leave - NO government sponsorship provided | After 2 years:   - Guaranteed paid study leave - NO government sponsorship provided |
| ***Housing*** | SUPERIOR HOUSING:   - 3 bedrooms - Electricity - Piped running water, - Self-contained master bedroom - Security reinforcements (fence, grills, bars on windows) | BASIC ALLOWANCE:   - No housing offered - 20% of Basic salary housing allowance provided |
| ***Transport*** | AVAILABLE: Reliable access to ambulance and utility vehicle (motorbike/ vehicle) for official facility use only | AVAILABLE: Reliable access to ambulance and utility vehicle (motorbike/ vehicle) for official facility use only |
| ***Facility equipment*** | INADEQUATE: Standard list of medical equipment at health facility NOT always available | ADEQUATE: Standard list of medical equipment at health facility always available |
| **Which posting would you choose?** | 🞏 | 🞏 |

| **JOB POSTING CHOICE SET #12** | | |
| --- | --- | --- |
|  | **Posting A** | **Posting B** |
| ***Location*** | Rural | Urban |
| ***Salary*** | Basic salary | Basic salary |
| ***Educational opportunities*** | After 2 years:   - Guaranteed paid study leave - NO government sponsorship provided | After 2 years:   - Guaranteed paid study leave - NO government sponsorship provided |
| ***Housing*** | BASIC ALLOWANCE:   - No housing offered - 20% of Basic salary housing allowance provided | SUPERIOR HOUSING:   - 3 bedrooms - Electricity - Piped running water, - Self-contained master bedroom - Security reinforcements (fence, grills, bars on windows) |
| ***Transport*** | AVAILABLE: Reliable access to ambulance and utility vehicle (motorbike/ vehicle) for official facility use only | AVAILABLE: Reliable access to ambulance and utility vehicle (motorbike/ vehicle) for official facility use only |
| ***Facility equipment*** | ADEQUATE: Standard list of medical equipment at health facility always available | ADEQUATE: Standard list of medical equipment at health facility always available |
| **Which posting would you choose?** | 🞏 | 🞏 |

**BLOCK 2**

| **PART 1. DEMOGRAPHIC QUESTIONS** | |
| --- | --- |
| *Instructions: Please answer the following questions to the best of your ability.* | |
| 1. **What is your gender?** | 🞏 Male  🞏 Female |
| 1. **What is your age group?** | 🞏 19 years or younger  🞏 20 to 29 years  🞏 30 to 39 years  🞏 40 to 49 years  🞏 50 years or older |
| 1. **What is your marital status?** | 🞏 Single  🞏 Engaged  🞏 Married  🞏 Divorced or widowed |
| 1. **Do you have any dependents that live with you?** | 🞏 Yes  🞏 No |
| 1. **Please indicate which age group(s) your children fall into. (Check all that apply)** | 🞏 Under 2 years  🞏 2 to 6 years  🞏 7 to 12 years  🞏 13 to 18 years  🞏 Older than 18 years  🞏 Not applicable |
| 1. **Have you at any time lived in a rural area for more than 1 year at a time?** | 🞏 Yes  🞏 No |

| **PART 2. PROFESSIONAL BACKGROUND QUESTIONS** | |
| --- | --- |
| *Instructions: Please answer the following questions to the best of your ability.* | |
| 1. **Are you a currently a Student or a Health Care Worker** | 🞏 Health Care Worker  🞏 Student (Pre-service)  🞏 Student (In-service) |
| 1. **Indicate your professional cadre** | 🞏 Enrolled Nurse  🞏 Registered Nurse  🞏 Enrolled Midwife  🞏 Registered Midwife  🞏 Environmental Health Technologist  🞏 Clinical Officer  🞏 Medical Licentiate  🞏 Medical Doctor  🞏 Other __________________ |
| 1. **Has your experience been in the public or the private sector?** | 🞏 All public sector  🞏 Both public and private sector  🞏 All private sector  🞏 Not applicable |
| 1. **How many years have you worked as a health worker?** | 🞏 Less than 1 year  🞏 1 to 3 years  🞏 4 to 6 years  🞏 7 to 10 years  🞏 11 or more years  🞏 Not applicable |
| 1. **How many years have you worked as a health worker in rural areas?** | 🞏 Less than 1 year  🞏 1 to 3 years  🞏 4 to 6 years  🞏 7 to 10 years  🞏 11 or more years  🞏 Not applicable |
| 1. **Please rate your overall experience working in rural areas?** | 🞏 Excellent or very good  🞏 Good  🞏 Fair  🞏 Poor  🞏 I have never worked in a rural area |
| 1. **Are you under any obligation or have you made any commitment to work in a rural area for a certain amount of time or in exchange for some benefit?** | 🞏 Yes  🞏 No  If **YES** please indicate the type of benefit________________________________ |
| 1. **Are you planning to upgrade your qualifications in the future?** | 🞏 Yes  🞏 No  🞏 Not decided |
| 1. **Please rate how likely you are to work in a rural area in the next 5 years.** | 🞏 Very likely  🞏 Likely  🞏 Unlikely  🞏 Very unlikely |

| **PART 3. JOB POSTING CHOICE SETS** |
| --- |
| *Instructions:*   - *Imagine that you have applied for a job or transfer of posting, and you have been given options about which job you would prefer to take.* - *In each of the following choice sets, please indicate whether you would be more likely to accept Job Posting A or Job Posting B, based on the information provided.* - *Job A is always in a rural location and Job B always is in an urban location.* |

**An example of basic salary with percentage allowances**

| **Basic salary (per month)** | **20% allowance** | **25% allowance** | **30% allowance** |
| --- | --- | --- | --- |
| **K2,800** | **K560** | **K700** | **K840** |
| **K3,200** | **K640** | **K800** | **K960** |
| **K3,500** | **K700** | **K875** | **K1,050** |
| **K7,376** | **K1,475** | **K1,844** | **K2,213** |

| **JOB POSTING CHOICE SET #1** | | |
| --- | --- | --- |
|  | **Posting A** | **Posting B** |
| ***Location*** | Rural | Urban |
| ***Salary*** | Basic salary + 25% rural allowance | Basic salary |
| ***Educational opportunities*** | After 3 years:   - Guaranteed paid study leave - Eligible (not guaranteed) for 75% government sponsorship | After 2 years:   - Guaranteed paid study leave - NO government sponsorship provided |
| ***Housing*** | BASIC HOUSING:   - 2 bedrooms - Outside bathroom - No electricity - Water available through bore hole or hand pump | SUPERIOR HOUSING:   - 3 bedrooms - Electricity - Piped running water - Self-contained master bedroom - Security reinforcements (fence, grills, bars on windows) |
| ***Transport*** | NONE: No access to ambulance no utility vehicle | AVAILABLE: Reliable access to ambulance and utility vehicle (motorbike/ vehicle) for official facility use only |
| ***Facility equipment*** | INADEQUATE: Standard list of medical equipment at health facility NOT always available | ADEQUATE: Standard list of medical equipment at health facility always available |
| **Which posting would you choose?** | 🞏 | 🞏 |

| **JOB POSTING CHOICE SET #2** | | |
| --- | --- | --- |
|  | **Posting A** | **Posting B** |
| ***Location*** | Rural | Urban |
| ***Salary*** | Basic salary + 30% rural allowance | Basic salary |
| ***Educational opportunities*** | After 4 years:   - Paid study leave guaranteed - 100% government sponsorship guaranteed | After 2 years:   - Guaranteed paid study leave - NO government sponsorship provided |
| ***Housing*** | BASIC HOUSING:   - 2 bedrooms - Outside bathroom - No electricity - Water available through bore hole or hand pump | BASIC HOUSING:   - 2 bedrooms - Outside bathroom - No electricity - Water available through bore hole or hand pump |
| ***Transport*** | AVAILABLE: Reliable access to ambulance and utility vehicle (motorbike/ vehicle) for official facility use only | AVAILABLE: Reliable access to ambulance and utility vehicle (motorbike/ vehicle) for official facility use only |
| ***Facility equipment*** | INADEQUATE: Standard list of medical equipment at health facility NOT always available | INADEQUATE: Standard list of medical equipment at health facility NOT always available |
| **Which posting would you choose?** | 🞏 | 🞏 |

| **JOB POSTING CHOICE SET #3** | | |
| --- | --- | --- |
|  | **Posting A** | **Posting B** |
| ***Location*** | Rural | Urban |
| ***Salary*** | Basic salary + 25% rural allowance | Basic salary |
| ***Educational opportunities*** | After 2 years:   - Guaranteed paid study leave - NO government sponsorship provided | After 2 years:   - Guaranteed paid study leave - NO government sponsorship provided |
| ***Housing*** | BASIC ALLOWANCE:   - No housing offered - 20% of Basic salary housing allowance provided | BASIC HOUSING:   - 2 bedrooms - Outside bathroom - No electricity - Water available through bore hole or hand pump |
| ***Transport*** | NONE: No access to ambulance no utility vehicle | NONE: No access to ambulance no utility vehicle |
| ***Facility equipment*** | INADEQUATE: Standard list of medical equipment at health facility NOT always available | INADEQUATE: Standard list of medical equipment at health facility NOT always available |
| **Which posting would you choose?** | 🞏 | 🞏 |

| **JOB POSTING CHOICE SET #4** | | |
| --- | --- | --- |
|  | **Posting A** | **Posting B** |
| ***Location*** | Rural | Urban |
| ***Salary*** | Basic salary + 20% rural allowance | Basic salary |
| ***Educational opportunities*** | After 3 years:   - Guaranteed paid study leave - Eligible (not guaranteed) for 75% government sponsorship | After 2 years:   - Guaranteed paid study leave - NO government sponsorship provided |
| ***Housing*** | BASIC ALLOWANCE:   - No housing offered - 20% of Basic salary housing allowance provided | BASIC ALLOWANCE:   - No housing offered - 20% of Basic salary housing allowance provided |
| ***Transport*** | NONE: No access to ambulance no utility vehicle | AVAILABLE: Reliable access to ambulance and utility vehicle (motorbike/ vehicle) for official facility use only |
| ***Facility equipment*** | ADEQUATE: Standard list of medical equipment at health facility always available | INADEQUATE: Standard list of medical equipment at health facility NOT always available |
| **Which posting would you choose?** | 🞏 | 🞏 |

| **JOB POSTING CHOICE SET #5** | | |
| --- | --- | --- |
|  | **Posting A** | **Posting B** |
| ***Location*** | Rural | Urban |
| ***Salary*** | Basic salary + 25% rural allowance | Basic salary |
| ***Educational opportunities*** | After 4 years:   - Paid study leave guaranteed - 100% government sponsorship guaranteed | After 2 years:   - Guaranteed paid study leave - NO government sponsorship provided |
| ***Housing*** | BASIC HOUSING:   - 2 bedrooms - Outside bathroom - No electricity - Water available through bore hole or hand pump | BASIC ALLOWANCE:   - No housing offered - 20% of Basic salary housing allowance provided |
| ***Transport*** | AVAILABLE: Reliable access to ambulance and utility vehicle (motorbike/ vehicle) for official facility use only | AVAILABLE: Reliable access to ambulance and utility vehicle (motorbike/ vehicle) for official facility use only |
| ***Facility equipment*** | ADEQUATE: Standard list of medical equipment at health facility always available | INADEQUATE: Standard list of medical equipment at health facility NOT always available |
| **Which posting would you choose?** | 🞏 | 🞏 |

| **JOB POSTING CHOICE SET #6** | | |
| --- | --- | --- |
|  | **Posting A** | **Posting B** |
| ***Location*** | Rural | Urban |
| ***Salary*** | Basic salary | Basic salary |
| ***Educational opportunities*** | After 4 years:   - Paid study leave guaranteed - 100% government sponsorship guaranteed | After 2 years:   - Guaranteed paid study leave - NO government sponsorship provided |
| ***Housing*** | SUPERIOR ALLOWANCE:   - No housing offered - 30% of Basic salary housing allowance provided | BASIC HOUSING:   - 2 bedrooms - Outside bathroom - No electricity - Water available through bore hole or hand pump |
| ***Transport*** | NONE: No access to ambulance no utility vehicle | AVAILABLE: Reliable access to ambulance and utility vehicle (motorbike/ vehicle) for official facility use only |
| ***Facility equipment*** | INADEQUATE: Standard list of medical equipment at health facility NOT always available | ADEQUATE: Standard list of medical equipment at health facility always available |
| **Which posting would you choose?** | 🞏 | 🞏 |

| **JOB POSTING CHOICE SET #7** | | |
| --- | --- | --- |
|  | **Posting A** | **Posting B** |
| ***Location*** | Rural | Urban |
| ***Salary*** | Basic salary + 30% rural allowance | Basic salary |
| ***Educational opportunities*** | After 4 years:   - Paid study leave guaranteed - 100% government sponsorship guaranteed | After 2 years:   - Guaranteed paid study leave - NO government sponsorship provided |
| ***Housing*** | BASIC ALLOWANCE:   - No housing offered - 20% of Basic salary housing allowance provided | BASIC ALLOWANCE:   - No housing offered - 20% of Basic salary housing allowance provided |
| ***Transport*** | NONE: No access to ambulance no utility vehicle | NONE: No access to ambulance no utility vehicle |
| ***Facility equipment*** | ADEQUATE: Standard list of medical equipment at health facility always available | ADEQUATE: Standard list of medical equipment at health facility always available |
| **Which posting would you choose?** | 🞏 | 🞏 |

| **JOB POSTING CHOICE SET #8** | | |
| --- | --- | --- |
|  | **Posting A** | **Posting B** |
| ***Location*** | Rural | Urban |
| ***Salary*** | Basic salary + 20% rural allowance | Basic salary |
| ***Educational opportunities*** | After 4 years:   - Paid study leave guaranteed - 100% government sponsorship guaranteed | After 2 years:   - Guaranteed paid study leave - NO government sponsorship provided |
| ***Housing*** | SUPERIOR HOUSING:   - 3 bedrooms - Electricity - Piped running water - Self-contained master bedroom - Security reinforcements (fence, grills, bars on windows) | SUPERIOR HOUSING:   - 3 bedrooms - Electricity - Piped running water - Self-contained master bedroom - Security reinforcements (fence, grills, bars on windows) |
| ***Transport*** | NONE: No access to ambulance no utility vehicle | NONE: No access to ambulance no utility vehicle |
| ***Facility equipment*** | INADEQUATE: Standard list of medical equipment at health facility NOT always available | ADEQUATE: Standard list of medical equipment at health facility always available |
| **Which posting would you choose?** | 🞏 | 🞏 |

| **JOB POSTING CHOICE SET #9** | | |
| --- | --- | --- |
|  | **Posting A** | **Posting B** |
| ***Location*** | Rural | Urban |
| ***Salary*** | Basic salary + 25% rural allowance | Basic salary |
| ***Educational opportunities*** | After 4 years:   - Paid study leave guaranteed - 100% government sponsorship guaranteed | After 2 years:   - Guaranteed paid study leave - NO government sponsorship provided |
| ***Housing*** | SUPERIOR HOUSING:   - 3 bedrooms - Electricity - Piped running water - Self-contained master bedroom - Security reinforcements (fence, grills, bars on windows) | SUPERIOR HOUSING:   - 3 bedrooms - Electricity - Piped running water - Self-contained master bedroom - Security reinforcements (fence, grills, bars on windows) |
| ***Transport*** | NONE: No access to ambulance no utility vehicle | AVAILABLE: Reliable access to ambulance and utility vehicle (motorbike/ vehicle) for official facility use only |
| ***Facility equipment*** | ADEQUATE: Standard list of medical equipment at health facility always available | ADEQUATE: Standard list of medical equipment at health facility always available |
| **Which posting would you choose?** | 🞏 | 🞏 |

| **JOB POSTING CHOICE SET #10** | | |
| --- | --- | --- |
|  | **Posting A** | **Posting B** |
| ***Location*** | Rural | Urban |
| ***Salary*** | Basic salary | Basic salary |
| ***Educational opportunities*** | After 4 years:   - Paid study leave guaranteed - 100% government sponsorship guaranteed | After 2 years:   - Guaranteed paid study leave - NO government sponsorship provided |
| ***Housing*** | BASIC ALLOWANCE:   - No housing offered - 20% of Basic salary housing allowance provided | SUPERIOR HOUSING:   - 3 bedrooms - Electricity - Piped running water - Self-contained master bedroom - Security reinforcements (fence, grills, bars on windows) |
| ***Transport*** | AVAILABLE: Reliable access to ambulance and utility vehicle (motorbike/ vehicle) for official facility use only | NONE: No access to ambulance no utility vehicle |
| ***Facility equipment*** | INADEQUATE: Standard list of medical equipment at health facility NOT always available | INADEQUATE: Standard list of medical equipment at health facility NOT always available |
| **Which posting would you choose?** | 🞏 | 🞏 |

| **JOB POSTING CHOICE SET #11** | | |
| --- | --- | --- |
|  | **Posting A** | **Posting B** |
| ***Location*** | Rural | Urban |
| ***Salary*** | Basic salary | Basic salary |
| ***Educational opportunities*** | After 2 years:   - Guaranteed paid study leave - NO government sponsorship provided | After 2 years:   - Guaranteed paid study leave - NO government sponsorship provided |
| ***Housing*** | BASIC ALLOWANCE:   - No housing offered - 20% of Basic salary housing allowance provided | BASIC HOUSING:   - 2 bedrooms - Outside bathroom - No electricity - Water available through bore hole or hand pump |
| ***Transport*** | NONE: No access to ambulance no utility vehicle | NONE: No access to ambulance no utility vehicle |
| ***Facility equipment*** | ADEQUATE: Standard list of medical equipment at health facility always available | ADEQUATE: Standard list of medical equipment at health facility always available |
| **Which posting would you choose?** | 🞏 | 🞏 |

| **JOB POSTING CHOICE SET #12** | | |
| --- | --- | --- |
|  | **Posting A** | **Posting B** |
| ***Location*** | Rural | Urban |
| ***Salary*** | Basic salary +20% rural allowance | Basic salary |
| ***Educational opportunities*** | After 2 years:   - Guaranteed paid study leave - NO government sponsorship provided | After 2 years:   - Guaranteed paid study leave - NO government sponsorship provided |
| ***Housing*** | SUPERIOR ALLOWANCE:   - No housing offered - 30% of Basic salary housing allowance provided | SUPERIOR HOUSING:   - 3 bedrooms - Electricity - Piped running water - Self-contained master bedroom - Security reinforcements (fence, grills, bars on windows) |
| ***Transport*** | AVAILABLE: Reliable access to ambulance and utility vehicle (motorbike/ vehicle) for official facility use only | AVAILABLE: Reliable access to ambulance and utility vehicle (motorbike/ vehicle) for official facility use only |
| ***Facility equipment*** | ADEQUATE: Standard list of medical equipment at health facility always available | INADEQUATE: Standard list of medical equipment at health facility NOT always available |
| **Which posting would you choose?** | 🞏 | 🞏 |
