# Supplementary material for: Assessment of interventions to attract and retain health workers in rural Zambia: a discrete choice experiment
Source: Hum Resour Health. 2019 Apr 3;17:26. doi: 10.1186/s12960-019-0359-3 (PMC6448309; doi:10.1186/s12960-019-0359-3)
Supplement: Supplementary file 2 — Costing detail. (DOCX 37 kb) [file 12960_2019_359_MOESM2_ESM.docx]

**Additional file 2: Costing detail**

In order to consider the job attribute options in the context of government policy, it is important to understand the costs of each option included in a DCE questionnaire. In order to estimate the costs of each option, we followed several key principles:

1. **Reasonable expectation**: The descriptions provided in the DCE included some detail about each job attribute, but we were required to make some further assumptions in order to develop costs. In doing so, we aimed to cost what a reasonable participant would have expected to receive based on the description given in the questionnaire.
2. **Total and marginal costs**: We have considered the cost first in terms of the actual total costs that would be required to implement any given level of an attribute. Additionally, because the results of the DCE questionnaire are framed in terms of the increased odds of taking a job if a strategy were implemented compared to the base level, we also estimated the *additional or marginal cost* of implementing the strategy compared to the cost of the base level.
3. **Cost per working year standardization**: In order to standardize the way that costs are represented we calculated the cost per working year of offering a given strategy. For strategies that require annual provision (like a rural allowance) this is very straightforward. For costs such as a house, which is built one at a time but used over a certain period, we needed to make some assumptions in order to standardize the cost to be represent a per-working-year cost.
4. **Costs for registered nurses**: In the case of some strategies, the costs would vary based on the health worker cadre (for example, due to differences in base salaries or years of schooling required to upgrade). We performed this cost exercise using registered nurses because 1) registered nurses make up a large proportion of the work force, 2) registered nurses are positioned in the mid-range or the salary scale (lower than midwives and doctors) but higher than EHTs and enrolled nurses, 3) the salary for registered nurses is the same as that for enrolled midwives and clinical officers (though upgrading requirements may vary slightly).

Based on these principles, we developed marginal cost estimates per working year for each of the job attributes relative to the base level within that attribute category. These costs and the other assumptions associated with calculations for each attribute category are shown in **Table A** and the calculations steps and details are shown in **Table B**.

**Table A. Cost estimates for job attributes in DCE questionnaire**

| **Job attribute** | **Total cost** | | **Marginal cost (compared to base)** | | **Assumptions** |
| --- | --- | --- | --- | --- | --- |
|  | **ZMW** | **USD^1^** | **ZMW** | **USD^1^** |  |
| **SALARY** |  |  |  |  |  |
| Base salary | 66,576 | $ 7,084 | - | - | Base salary is ZMW 43,800. Full salary inclusive of the following allowances: 10% transport allowance, 20% housing allowance, 15% health shift allowance, 7% commuted night duty, and rural allowances as stated. This figure represents a gross salary (before taxes). |
| Base salary + 20% rural allowance | 75,336 | $ 8,016 | 8,760 | $ 932 |  |
| Base salary + 25% rural allowance | 77,526 | $ 8,249 | 10,950 | $ 1,165 |  |
| Base salary + 30% rural allowance | 79,716 | $ 8,482 | 13,140 | $ 1,398 |  |
| **HOUSING** |  |  |  |  |  |
| Basic allowance offered (20% of base salary), but no housing provided | 8,760 | $ 932 | - | - | For basic and superior housing, the houses would be constructed for $20,000 and $45,000, respectively and would be occupied for 25 years. These housing cost values are based on input from the MOH and Solidaire Med on the average costs of housing constructed in rural areas of Zambia. We assume that the standard 20% housing allowance would still be collected, but would be used to go into a fund for housing maintenance following a model used by Solidaire Med. |
| Basic housing provided (2 bedrooms, outside bathroom, no electricity, water available through bore hole or hand pump) | 16,278 | $ 1,732 | 7,518 | $ 800 |  |
| Superior housing provided (3 bedrooms, electricity, piped running water, self-contained master bedroom and security reinforcements) | 25,676 | $ 2,732 | 16,916 | $ 1,800 |  |
| Superior allowance offered (30% of base salary), but no housing provided | 13,140 | $ 1,398 | 4,380 | $ 466 |  |
| **EDUCATION** |  |  |  |  |  |
| Guaranteed paid leave after two years with no government financial assistance for study | 85,410 | $ 9,088 | - | - | While a student is on study leave, we assume that their salary would still be paid (inclusive of transport and housing allowances). The salary while in school is divided over the working years before school leave to estimate the marginal cost of retaining a year worker per year. School leave would last for 3 years and a full scholarship would cost ZMW 60,000 per year, based on scholarship standards from the Ministry of Health for registered nursing programs. For the scenario with a chance of a scholarship we assumed that 60% of health workers would get the scholarship. |
| Guaranteed paid leave after three years with eligibility for (not guaranteed) 75% government scholarship for study | 83,940 | $ 8,932 | - 1,470 | -$ 156 |  |
| Guaranteed paid leave after four years with 100% government scholarship for study guaranteed | 87,705 | $ 9,332 | 2,295 | $ 244 |  |
| **TRANSPORT** |  |  |  |  |  |
| No access to ambulance or utility vehicle | - | - | - | - | Vehicle purchase cost would be $35,000 and annual maintenance would be $5,000, based on program records from CHAI programs in rural Zambia and confirmed by government expert input. The vehicle would be in use for 10 years and would benefit 2 employees. |
| Reliable access to ambulance and utility vehicle (motorbike/ vehicle) for official facility use only | 39,941 | $ 4,250 | 39,941 | $ 4,250 |  |
| **MEDICAL EQUIPMENT** |  |  |  |  |  |
| Standard list of medical equipment at health facility NOT always available | - | - | - | - | Initial investment to upgrade a health center would be $20,000 and annual maintenance would be $3,000, based on expert input from the Ministry of Health. The initial improvements would be sound for 6 years and would benefit 2 employees. |
| Standard list of medical equipment at health facility always available | 29,760 | $ 3,167 | 29,760 | $ 3,167 |  |

- - 1. The exchange rate used was 1 USD to 9.3979 ZMW, which represents the average rate from January through October 2017.
    2. All costing estimates calculated with assistance from the Ministry of Health

**Table B. Calculation detail for cost estimates**

|  | **ZMW** | **USD** |
| --- | --- | --- |
| **SALARY** |  |  |
|  |  |  |
| ***Base salary*** |  |  |
| ***Current Net Salary (Registered nurses)*** |  |  |
| Base salary | 43,800 | $ 4,661 |
| Transport: 10% | 4,380 | $ 466 |
| Housing: 20% | 8,760 | $ 932 |
| Commuted night duty: 7% (not offered in study leave) | 3,066 | $ 326 |
| Health shift allowance: 15% (not offered in study leave) | 6,570 | $ 699 |
| Total | 66,576 | $ 7,084 |
|  |  |  |
| ***With 20% rural allowance*** |  |  |
| Allowance | 20% |  |
| Allowance amount | 8,760 | $ 932 |
| Salary with allowance | 75,336 | $ 8,016 |
| Marginal increase over base | 8,760 | $ 932 |
|  |  |  |
| ***With 25% rural allowance*** |  |  |
| Allowance | 25% |  |
| Allowance amount | 10,950 | $ 1,165 |
| Salary with allowance | 77,526 | $ 8,249 |
| Marginal increase over base | 10,950 | $ 1,165 |
|  |  |  |
| ***With 30% rural allowance*** |  |  |
| Allowance | 30% |  |
| Allowance amount | 13,140 | $ 1,398 |
| Salary with allowance | 79,716 | $ 8,482 |
| Marginal increase over base | 13,140 | $ 1,398 |
|  |  |  |
| **HOUSING** |  |  |
|  |  |  |
| ***Basic allowance*** |  |  |
| Base salary | 43,800 | $ 4,661 |
| 20% allowance | 8,760 | $ 932 |
| Cost per year | 8,760 | $ 932 |
|  |  |  |
| ***Superior allowance*** |  |  |
| 30% allowance | 13,140 | $ 1,398 |
| Cost per health worker year | 13,140 | $ 1,398 |
| Marginal increase over base | 4,380 | $ 466 |
|  |  |  |
| ***Basic housing*** |  |  |
| Cost of house | 187,959 | $ 20,000 |
| Life of house | 25 | years |
| Maintenance cost per year (20% allowance) | 8,760 | $ 932 |
| Cost per health worker year | 16,278 | $ 1,732 |
| Marginal increase over base | 7,518 | $ 800 |
|  |  |  |
| ***Superior housing*** |  |  |
| Cost of house | 422,907 | $ 45,000 |
| Life of house | 25 | years |
| Maintenance cost per year (20% allowance) | 8,760 | $ 932 |
| Cost per health worker year | 25,676 | $ 2,732 |
| Marginal increase over base | 16,916 | $ 1,800 |
|  |  |  |
| **EDUCATION** |  |  |
|  |  |  |
| ***After 2 years, no scholarship*** |  |  |
| Salary while out per year | 56,940 | $ 6,059 |
| Length of school program | 3 | years |
| Salary while out (total) | 170,820 | $ 18,176 |
| Time before scholarship | 2 | years |
| Cost per health worker year | 85,410 | $ 9,088 |
|  |  |  |
| ***After 3 years, chance of 75% scholarship*** |  |  |
| Salary while out (total) | 170,820 | $ 18,176 |
| Cost of scholarship per year | 45,000 | $ 4,788 |
| Length of scholarship | 3 | years |
| Time before scholarship | 3 | years |
| % of students getting scholarship | 60% |  |
|  |  |  |
| Cost per health worker year | 83,940 | $ 8,932 |
| Marginal increase over base | -1,470 | $ -156 |
|  |  |  |
| ***After 4 years, guaranteed 100% scholarship*** |  |  |
| Salary while out (total) | 170,820 | $ 18,176 |
| Cost of scholarship per year | 60,000 | $ 6,384 |
| Length of scholarship | 3 | years |
| Time before scholarship | 4 | years |
| Percentage of students getting scholarship | 100% |  |
|  |  |  |
| Cost per health worker year | 87,705 | $ 9,332 |
| Marginal increase over base | 2,295 | $ 244 |
|  |  |  |
| **TRANSPORT** |  |  |
|  |  |  |
| ***Ambulance and utility vehicle*** |  |  |
| Cost of vehicle | 328,928 | $ 35,000 |
| Life of vehicle | 10 | years |
| Employees to benefit | 2 |  |
| Maintenance per year | 46,990 | $ 5,000 |
| Cost per person per year | 39,941 | $ 4,250 |
|  |  |  |
| **MEDICAL EQUIPMENT** |  |  |
|  |  |  |
| ***Making standard list of equipment available*** |  |  |
| Initial cost at health center | 187,959 | $ 20,000 |
| Initial cost at health post | 75,183 | $ 8,000 |
| Life of improvements | 6 | years |
| Employees to benefit | 2 |  |
| maintenance per year | 28,194 | $ 3,000 |
| Cost per person per year | 29,760 | $ 3,167 |
